# Impacts of Poor Social Support on General Health Status in Community-Dwelling Korean Elderly: The Results from the Korean Longitudinal Study on Health and Aging

**DOI:** 10.4306/pi.2008.5.3.155

**Published:** 2008-09-30

**Authors:** Jae Kyung Shin, Ki Woong Kim, Joon Hyuk Park, Jung Jae Lee, Yoonseok Huh, Seok Bum Lee, Eun Ae Choi, Dong Young Lee, Jong Inn Woo

**Affiliations:** 1 Department of Neuropsychiatry, Seoul National University Hospital, Seoul, Korea.; 2 Department of Neuropsychiatry, Seoul National University Bundang Hospital, Seongnam, Korea.; 3 Department of Psychiatry, Seoul National University College of Medicine, Seoul, Korea.; 4 Gongju National Hospital, Gongju, Korea.

**Keywords:** Social support, General health, Geriatric depression, Quality of life, Elderly

## Abstract

**Objective:**

We investigated the influence of social support on health, quality of life (QOL), and the risk of depression in elderly Korean people.

**Methods:**

This study was conducted as a part of the Korean Longitudinal Study on Health and Aging (KLoSHA). A total of 787 nondemented community-dwelling elderly aged 65 years or older were recruited and underwent clinical evaluations for dementia and psychiatric disorders conformed to Korean version of the Consortium to Establish a Registry for Alzheimer's Disease Clinical Assessment Battery (CERAD-K) and the Korean version of the Mini-International Neuropsychiatric Interview (MINI), respectively. Social support was assessed using the Medical Outcome Study Social Support Survey (MOS-SSS). Poor social support (PSS) was defined as having a MOS-SSS score below the 25^th^ percentile of the entire sample. General health status was comprehensively evaluated using the modified Cumulative Illness Rating Scale (CIRS), the Korean version of the Geriatric Depression Scale (GDS-K), Mini-Mental Status Examination (MMSE-KC), Korean Activities of Daily Living (KADL), and Korean Instrumental Activities of Daily Living (KIADL). Health-related QOL was evaluated using the Short Form 36 (SF-36).

**Results:**

Low educational attainment and living alone were associated with PSS. Geriatric depression was more prevalent in the PSS group (OR=3.05, 95% CI=1.77-5.27) than in the normal social support (NSS) group. Among the various forms of social support, positive social interaction was significantly associated with risk of geriatric depression (OR=2.25, 95% CI=1.07-4.73). Although health-related QOL was lower in the PSS group than in the NSS group, the ADL and IADL scores of the subjects in the PSS group were better than those of the subjects in the NSS group. In the subjects with geriatric depression, PSS was associated with more severe depression, higher medical morbidity, and poor QOL.

**Conclusion:**

PSS had a negative influence on the general health status and QOL among community-dwelling elderly and was an independent risk factor of geriatric depression.

## Introduction

Social support that includes emotional support as well as instrumental support is a coping resource.^1^,^2^ It's role in maintaining an individual's health is growing with the modernization of the society.^3^-^5^ Social support has been reported to be a more a important factor in health promotion and mortality reduction in the later stages of life.^1^,^3^-^8^ As people age, they experience different kinds of social loss, including deaths of family members and job loss, and the ability to cope with such losses diminishes due to the decreased physical and cognitive functions.^4^,^9^ As a result, the influence of social support on health becomes greater in elderly populations.^4^,^9^

Geriatric depression, along with other chronic illnesses, is one of the most common health problems in late life. The influence of social support on the onset of geriatric depression and its management is critical.^1^,^9^,^10^ Geriatric depression is a serious condition that reduces one's quality of life, not only in terms of mental health, increasing suicide and reducing vitality, but also in terms of physical health, as it can aggravate physical illness and increase mortality.^1^,^11^,^12^ Yet it has a subsyndromal characteristic, and its symptoms do not satisfy the conventional diagnostic criteria of major depressive disorder,^13^ and thus many patients with geriatric depression are neglected.^12^,^13^ Social support plays a major role in this population of patients with subsyndromal depression.^14^ It protects elderly individuals from the devastating consequences of depression by buffering the negative psychosocial effects of stressors,^1^,^4^,^9^ decreasing the risk of newonset depression,^4^,^9^ or reducing suicidal ideation.^1^ Therefore, in the elderly, a good social support system can be an effective non-pharmacological intervention to reduce new-onset depression and improve depressive symptoms in patients with depression.

Korea is currently experiencing a rapid aging of its population, and geriatric depression is becoming more prevalent. But so far there has been no comprehensive study demonstrating the influence of social support on health, quality of life, the risk of geriatric depression and its characteristics in the Korean geriatric population. Therefore, the authors investigated the influence of social support on health, quality of life, the risk of depression and its characteristics in Korean elderly.

## Methods

### Subjects

The study subjects were selected from community-dwelling elderly aged 65 years or older who participated in the baseline study of the Korean Longitudinal Study on Health and Aging (KLoSHA),^15^ which was conducted from September 2005 to August 2006. A total of 1,118 persons living in Seongnam City were randomly selected from the roster of 65,436 persons aged 65 years or older, and 714 persons agreed to participate in the baseline study. For the study of the oldest old, 3,166 persons aged 85 years and older were invited to join the study, and 286 of them agreed to participate in the baseline study of the KLoSHA. The final sample consisted of 1,000 subjects, and all subjects were fully informed of the study protocol and provided written informed consent to participate in the study.

The subjects who had a history of cerebrovascular disorders other than transient ischemic attack, and who had several physical illnesses, which interfere with neuropsychological tests, based on clinical examinations were excluded from the study. Subjects with any major axis I psychiatric disorder according to the Diagnostic and Statistical Manual of Mental Disorders, fourth edition (DSM-IV), were also excluded.^16^

### Assessment

A psychiatric specialist evaluated all of the subjects for an axis I DSM-IV diagnosis using the Mini-International Neuropsychiatric Interview (MINI).^17^ A structured diagnostic evaluation on dementia was also conducted using the Korean version of the Consortium to Establish a Registry of Alzheimer's Disease (CERAD-K) assessment battery.^18^

The social support system of the subjects was assessed by the Medical Outcome Study Social Support Survey (MOS-SSS).^19^ The MOS-SSS is a self-administered, multidimensional instrument used to assess various aspects of functional social support, including 4 subcategories of emotional/information support, tangible support, positive social interaction and affectionate support. It is composed of 19 items, including 8 items on emotional/information support, 4 items on tangible support, 4 items on positive social interaction and 3 items on affectionate support. All raw item scores are transformed into a scale of 0 to 100, and higher scores are indicative of better social support. The subjects were divided into 2 groups according to their level of social support. Poor social support (PSS) was defined as having a MOS-SSS score below the 25^th^ percentile of the entire sample.^20^ Normal social support (NSS) was defined as having a MOS-SSS score above the 25^th^ percentile of the entire sample.

The total burden of medical illness was rated by the modified Cumulative Illness Rating Scale (CIRS),^21^ and the 'psychiatric illness' category was not included.

Major depressive disorder was diagnosed according to the DSM-IV criteria, and minor depressive disorder was diagnosed according to the research criteria proposed in appendix B of the DSM-IV criteria. Considering the subsyndromal characteristic, geriatric depression was defined to include both major depressive disorder and minor depressive disorder in this study. Severity of depressive symptoms was measured using the Korean version of the Geriatric Depression Scale (GDS-K)^22^ and the Korean version of the Hamilton Depression Rating Scale (HAM-D).^23^

Global cognitive functioning was evaluated using the Mini Mental Status Examination in the Korean version of CERAD assessment packet (MMSE-KC).^24^ The activities of daily living of the subjects were measured by the Korean Activities of Daily Living (KADL)^25^ and the Korean Instrumental Activities of Daily Living (KIADL).^26^ The quality of life was estimated by the Medical Outcome Study 36-item Short Form Health Survey Instrument (SF-36).^27^ Research neuropsychiatrists and trained psychiatric research nurses whose inter-rater reliabilities were confirmed in previous studies were responsible for all of the clinical ratings.^15^

### Statistical analysis

The characteristics of the subjects in the PSS and NSS groups were compared using Student's t-test for continuous variables and the chi-square test for categorical variables. The effect of social support on the risk of geriatric depression was analyzed by multivariate logistic regression analysis with age, gender, education level, cohabitation, monthly income and CIRS score as covariates. In the sub-sample of geriatric depression, analysis of covariance (ANCOVA) was used to compare the clinical characteristics of the subjects in the PSS and NSS groups. The level of significance was set at a p value of less than 0.05 for all analyses.

## Results

A total of 787 subjects completed the study. The NSS group was composed of 592 subjects, and the PSS group was composed of 195 subjects. The mean MOS-SSS total score was 78.46±14.92 in the NSS group and 35.46 ±13.08 in the PSS group (p<0.001). As shown Table 1, the subjects in the NSS group scored higher than those in the PSS group on all four subcategories of social support.

Table 2 compares the characteristics of the subjects in the PSS group and those of the subjects in the NSS group. The educational attainment of the subjects in the PSS group was lower than that of the subjects in the NSS group (PSS=6.58±5.13 years, NSS=8.06±5.85 years, p=0.001). A greater number of subjects in the PSS group were living alone in comparison to the NSS group (NSS=11.8%, PSS=26.7%, p<0.001). However, age, gender, and monthly income did not differ according to the presence of PSS.

The PSS group had lower KADL (PSS=7.05±0.25, NSS=7.13±0.86, p=0.040) and KIADL (PSS=12.16±3.46, NSS=13.00±4.43, p=0.007) scores than the NSS group.

Although the modified CIRS scores of the subjects in the PSS group were comparable to those of the subjects in the NSS group, the SF-36 overall index, which reflects health-related QOL, scores of the subjects in the PSS group was much lower than that of the subjects in the NSS group (PSS=54.04±18.55, NSS=65.25±18.26, p<0.001). This was the case in mental health factor (PSS=59.92±16.70, NSS=72.69±16.20, p<0.001) and physical health factor (PSS=50.13±22.00, NSS=60.29± 21.90, p<0.001). The MMSE-KC scores did not differ according to the presence of PSS.

**FIGURE 1:**
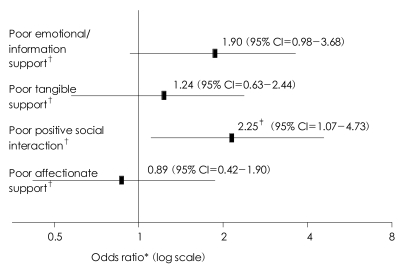
Risk of late-life depression conferred to poor social support. ^*^Multiple logistic regression analysis with adjustment for age, gender, education, cohabitation, low income with Enter method, ^†^Lower than 25^th^ percentile, ^‡^Significance<0.05.

Geriatric depression was more prevalent in the PSS group than in the NSS group (PSS=15.9%, NSS=5.7%, OR=3.05, 95% CI=1.77-5.27). The mean GDS-K (PSS=16.10±6.96, NSS=9.52±6.33, p<0.001) and HAM-D (PSS=5.43±4.84, NSS=3.31±3.36, p<0.001) scores were much higher in the PSS group than in the NSS group. Table 3 shows that poor social support was a siginificant risk factor of geriatric depression (OR=3.05, 95% CI=1.77-5.27). Positive social interaction was the only of the four subcategories of social support to be associated with the risk of geriatric depression. Poor positive social interaction increased the risk of geriatric depression by 2.25 times (95% CI=1.07-4.73, p=0.033) (Figure 1). Poor emotional/information support also increased the risk of geriatric depression, but this effect did not reach the level of statistical significance (OR=1.90, 95% CI=0.98-3.68, p=0.057).

**FIGURE 2:**
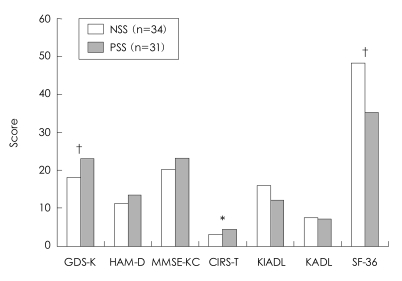
Influence of poor social support (PSS) on the clinical characteristics of patients with late-life depression. ^*^p<0.05, ^†^p<0.001, by ANCOVA adjusting age. GDS-K: Korean Geriatric Depression Scale, HAM-D: Hamilton Depression Rating scale, MMSEKC: Mini Mental Status Examination in the Korean version of CERAD Assessment Packet, CIRS-T: modified Cumulative Illness Rating Scale score, KIADL: Korean Instrumental Activities of Daily Living, KADL: Korean Activities of Daily Living, SF-36: Medical Outcome Study 36-item Short Form Health Survey Instrument.

In the patients with geriatric depression, 34 subjects belonged to the NSS group and 31 subjects belonged to the PSS group. The patients with geriatric depression in the PSS (GD-PSS) group were much younger than those in the NSS (GD-NSS) group (GD-PSS=73.35±8.23 years, GD-NSS=79.76±9.03 years, p=004). The GDS-K (GD-PSS=23.06±3.01, GD-NSS=18.18±6.92, p<0.001) and modified CIRS (GD-PSS=4.39±2.67, GD-NSS=3.06±2.08, p=0.037) scores of the subjects in the GD-PSS group were higher than those of the subjects in the GD-NSS group, indicating that PSS was associated with more severe subjective depressive symptoms and medical comorbidities in geriatric depression patients. The SF-36 score was also lower in the GD-PSS group than in the GD-NSS group (GD-PSS=35.21±18.18, GD-NSS=48.28±17.93, p<0.001)(Figure 2).

## Discussion

Differences between the NSS group and the PSS group were found in the patient scores on all 4 subcategories of social support and in the MOS-SSS total score. This finding is consistent with the finding that there was a high degree of correlation among the subcategories of the MOS-SSS, with correlation coefficients ranging from 0.69 to 0.82 when the MOS-SSS was first designed.^19^ The emotional/information support subcategory mainly covers empathetic understanding, emotional expression, advice and guidance. The positive social interaction subcategory involves sharing pleasurable activities, and the affectionate support category involves the expression of love. On the other hand, tangible support includes material aid and behavioral assistance. Some studies combined 3 subcategories other than tangible support into emotional support and simplified social support with 2 aspects.^28^,^29^

The subjects' scores on the tangible support subcategory were relatively high when compared with the other subcategories. The difference was about 8 points in the NSS group and 15 points in the PSS group. There has been no attempt to compare the MOS-SSS subcategory scores within a population. However, the outcome of the MOS-SSS development study was different than ours. There was little difference in scores on tangible support and other subcategories.^19^ However, the results of some Korean studies were similar to ours.^30^,^31^ In a study conducted in a population of Korean adult city dwellers of low economic status, there were about 10 points difference between MOS-SSS tangible support and overall index, both for men and women over the age of 60 years.^30^ Another study reported that instrumental support was higher than information support, emotional support and appraisal support in Korean elderly in rural areas.^31^ Tangible support provided to Korean elderly appears to be fine and satisfactory. However, with other areas of social support, Korean elderly experience shortages and dissatisfaction. Therefore, interventions and efforts to improve social support should be started in other areas of social support than tangible support.

A few studies have been conducted to clarify the effects of various sociodemographic factors on perceived functional social support in the elderly population. The previous studies only dealt with a few sociodemographic factors, if any at all,^28^,^32^-^34^ or they were conducted as a part of the study for the entire population.^5^,^30^,^32^,^35^

Some reported that elderly adults receive a higher level of social support than young or middle-aged adults.^30^,^36^ In this study, age had no effect on perceived level of social support. Age seems to have no additional effect on social support in the elderly population. We can postulate that the increase in the level of social support with age in previous studies is a reflection of the fact that elderly persons need more help from the family or community after retirement. Although gender differences in perceived social support have not been consistently reported,^4^,^5^,^28^,^30^,^32^,^37^ women had better social support than men in most Western countries.^4^,^5^,^32^ Our study did not reveal any effect of gender on social support. The influence of regional characteristics and economic status may confound the effects of gender on social support, resulting in inconsistent results.

In agreement with most of the earlier observations,^2^,^31^,^32^,^36^ cohabitation with a spouse or children was associated with better social support in the present study. However, most of the previous studies were concerned with 'being married' rather than 'cohabitation', and elderly individuals who never married showed better social support than those who were married in a previous study.^30^ The level and quality of social support may vary according to living arrangements. The elderly individuals who lived with their children received less support from their spouses, and those who lived with only their spouse received the least amount of support from their friends.^28^ Thus, the nature of social support seems to be multifactorial and complicated.

Higher educational attainment has been reported to be associated with better social support, as determined in our study,^2^,^30^-^32^,^34^ More education allows for increased access to different kinds of information^31^ and helps to expand the individual's social network.^2^,^32^,^34^ Like lower educational attainment, lower economic status has been known to be related with poorer social support.^2^,^30^-^32^,^34^ Although more subjects with lower economic status were in the PSS group in this study, statistical significance was not available. Korean elderly tend to rely on their children or other younger family members to meet their financial needs. However, the evaluation of economic status was only conducted on the basis of monthly income, and it could have led to overestimation of their economic difficulty. Economic status also affects network formation and accessibility to various resources and information.^34^ It is also reported that satisfaction with pocket money is correlated with a higher level of perceived social support.^31^ Thus, elderly individuals with low educational attainment and low economic status constitute a good starting point for the social support reinforcement program.

Poor social support is thought to be a risk factor for poor quality of life,^28^,^38^-^40^ and the findings of our study support this theory. The PSS group scored lower than the NSS group on assessments of both mental and physical health. It is not possible to determine the causal relationship behind these results from our data alone. Yet, it was postulated that functional social support has a direct effect on quality of life.^38^ In addition, in the vulnerable geriatric population, social network had a direct short-term effect on health status, but health status had no effect on social network.^7^ Thus, we believe that social support influences quality of life. Korean elderly are believed to have a family-based support system. However, many studies have indicated that social contact with friends and participation in club or church activities made a greater contribution to improved quality of life.^8^,^40^,^41^ Another study reported that affectionate support and positive social interaction had the most explanatory power on self-rated health status.^30^ It can be suggested that social support system should be extended and strengthened through the family system.

Decline in activities of daily living was more prominent in the NSS group than in the PSS group. This result was unexpected and inconsistent with common beliefs and other previous reports.^2^,^31^,^39^,^40^,^42^ As mentioned before, family is the center of the social support system for Korean elderly. Once an elderly person becomes disabled, familial support and care for the patient seem to increase. This is probably the reason for the contradictory result in our study. There was a similar observation that elderly individuals with higher IADL scores received better social support from children.^28^ However, there was no difference in CIRS score, which reflects the burden of general medical illness, between the two groups. The dual influence of social support and physical illness may have caused this contradiction. The correlation between social support and physical health seems to be counterbalanced because the elderly receiving more support due to physical health problems and the elderly suffering more health problems due to lack of social support were intermingled in the analysis. Therefore, further prospective study will be able to clarify the influence of social support on activities of daily living.

The influence of social support on geriatric depression has been extensively explored in the previous studies. Most of the previous studies demonstrated that social support decreases the risk of geriatric depression.^1^,^9^,^10^,^14^,^43^-^45^ However, the previous studies focused on functional social support apart from structural aspects. Few used comprehensive tools to evaluate its multidimensional aspect with sufficient consideration of sociodemographic and clinical factors. Few studies questioned which subcategory of social support would be most strongly related to geriatric depression. It is already accepted that emotional support has a greater influence on depressive symptoms late in life when compared with instrumental support.^46^ A strength of our study is that positive social interaction was identified to be the most influential form of social support. Supporting evidence for this finding can be easily found in studies concerning structural social support. Positive social interaction in the form of social contact plays a critical role in preventing geriatric depression.^1^,^9^,^44^ Others calculated the frequency of regular gatherings with friends and relatives or the number of close friends and demonstrated that some association between these factors and geriatric depression.^10^,^43^ This implies that social support essential for elders is not a simple concern or a physical care, but the establishment of social relationships by sharing leisure and pleasurable activities. Our consideration over the subsyndromal nature of geriatric depression is another strength of this study

In the sub-sample of patients with geriatric depression, the subjects in the PSS group were younger than those in the NSS group. This result is different from that of the total sample. The mean age (73.35±8.23 years) of the patients in the depressive PSS group was below 75 to 79 years, which Suh et al. reported as a risk factor for late-life depression in a Korean epidemiologic study.^47^ It is presumed that poor social support contributed to the early onset of geriatric depression. Even in the depressive sub-sample, the patients in the PSS group suffered more severe depressive symptoms and experienced a lower quality of life. This finding reaffirms the influence of social support on the quality of life and psychological distress, which is independent of depressive disorder itself.^38^ In contrast to the total sample, the patients in the depressive PSS group suffered a greater burden of medical comorbidities. It also reflects the reciprocal interaction of social support and physical illness as we discussed before. Geriatric depression is known to aggravate medical illness.^11^ It is reasonable to think that poor social support affected the severity of depression and finally worsened medical illness. Perceived social support was suggested to play a bridging role between depression and physical health status.^3^ Therefore, we can infer that improving social support in elderly patients with depression could reduce the burden of medical illness as well as depression itself.

A limitation of our cross-sectional study is that it cannot prove the causal relationship between social support and geriatric depression, but this will be solved in the ongoing prospective study. An association between social support and maintenance or recurrence of geriatric depression is also suspected.^10^ Future study should involve those aspects as well. Risks for suicidal ideation or suicide attempt are possible outcomes of geriatric depression, and fewer social relationships were reported to predict a greater risk of suicide.^1^ The role of social support in the prevention of suicide must be further investigated.

In this study, we confirmed the correlation between social support and geriatric depression and demonstrated that positive social interaction was a risk factor for geriatric depression among the subcategories of social support. The results suggest that psychosocial interventions are needed for the population experiencing the destruction of their social support system or suffering from geriatric depression, but with poor social support. At senior centers, adult day-care activities, self-help groups such as 'widow-to-widow' and volunteer organizations for elderly have been successfully applied to geriatric depression.^1^,^12^ Social support programs especially focusing on the establishment of social relationships among elderly individuals must be further investigated and developed based on the Korean situation.

## Figures and Tables

**TABLE 1:**
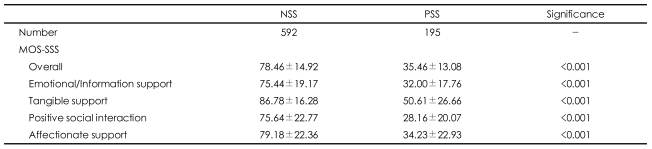
Comparison of social support status between the normal social support (NSS) group and the poor social support (PSS) group^*^

^*^PSS: Medical Outcome Study Social Support Survey (MOS-SSS) score <25 percentile

**TABLE 2:**
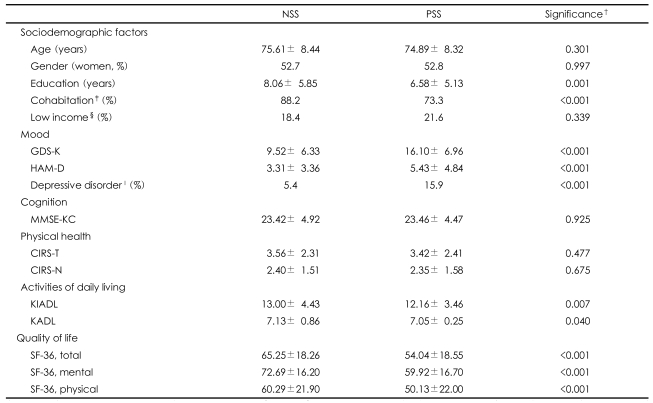
Comparison of sociodemographic and clinical characteristics between the normal social support (NSS) group and the poor social support (PSS) group^*^

^*^PSS: Medical Outcome Study Social Support Survey (MOS-SSS) score <25 percentile, ^†^Student t-tests for continuous variables and chi square tests for categorical variables, ^‡^Cohabit with his/her spouse or other family members, ^§^Income less than 12,000,000 KRW/year, ^∥^Depressive disorder=major depressive disorder+minor depressive disorder. GDS-K: Korean Geriatric Depression Scale, HAM-D: Hamilton Depression Rating Scale, MMSE-KC: Mini Mental Status Examination in the Korean version of the CERAD Assessment Packet, CIRS-T and CIRS-N: modified Cumulative Illness Rating Scale Total score and number of disease categories, KIADL: Korean Instrumental Activities of Daily Living, KADL: Korean Activities of Daily Living, SF-36: Medical Outcome Study 36-item Short Form Health Survey Instrument

**TABLE 3:**
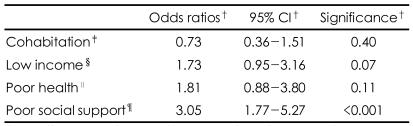
Social and clinical risk factors for late-life depression^*^

^*^Late-life depression=major depressive disorder+minor depressive disorder, ^†^Multiple logistic regression analysis adjusting age, gender, and education, ^‡^Cohabit with his/her spouse or other family members, ^§^Income less than 12,000,000 KRW/year, ^∥^Modified Cumulative Illness Rating Scale Total score higher than 1.5 SD of norm, ^¶^Medical Outcome Study Social Support Survey (MOS-SSS) score below the 25^th^ percentile
